# Microvesicle-Shuttled microRNA-130b Activates the Hepatic Inflammation by Inhibiting Glucocorticoid-Receptor-Mediated Immunosuppression in High-Fat Diet-Induced Obese Mice

**DOI:** 10.3390/vetsci11110565

**Published:** 2024-11-13

**Authors:** Zhengqiang Han, Lijun Wang, Shiyong Xu, Horsen Zhang, Ji Cheng, Shifeng Pan

**Affiliations:** 1 College of Animal Science and Food Engineering, Jinling Institute of Technology, Nanjing 210038, China; hzq@jit.edu.cn (Z.H.); xushiyong@jit.edu.cn (S.X.); 2 College of Veterinary Medicine, Yangzhou University, Yangzhou 225009, China; wlj6379@163.com (L.W.); 008743@yzu.edu.cn (J.C.); 3 Lesaffre (Mingguang) Co., Ltd., Chuzhou 239000, China; h.zhang@phileo.lesaffre.com; 4 Jiangsu Co-Innovation Center for Prevention and Control of Important Animal Infectious Diseases and Zoonoses, Yangzhou University, Yangzhou 225009, China

**Keywords:** microvesicles, miRNA-130b, hepatic inflammation, GR-mediated immunosuppression, high-fat diet-induced obese mice

## Abstract

Metabolism-disorder-induced liver diseases have become increasingly prevalent worldwide and are clinically linked to obesity and type 2 diabetes. In addition, a large number of previous literature studies have indicated that plasma miR-130b is a promising biomarker for the early diagnosis and treatment of obesity. However, whether miRNA-130b that was positively correlated with obesity resulted in hepatic inflammation needs to be further studied. Therefore, the study aims to determine the effect of microvesicle-shuttled miRNA-130b (miR-130b-MV) on the hepatic inflammation and its potential mechanism in high-fat diet-induced obese mice. Three-week-old C57BL/6 mice were fed a high-fat diet for eight weeks. Then, the obese mice received tail vein injections of MV-packaged scrambled control microRNA (miR-SC-MV) or miR-130b-MV every other day for 10 days. Compared with the control group, the miR-130b-MV injection significantly reduced the body weight while increasing the ratio of liver wet weight to total body weight. In addition, the miR-130b-MV injection significantly activated the hepatic inflammation by increasing the expression of proinflammatory genes, although the plasma concentrations of IL-6 and TNF-α were only slightly increased. Furthermore, the miR-130b-MV injection significantly increased the hepatic miR-130b expression while significantly suppressing the protein expression and phosphorylation of GR, a potential target of miR-130b. Moreover, the miR-130b overexpression results in a decrease in the expression of endogenous GR protein and a decrease in the activity of the luciferase reporter of GR 3′-UTR. In addition, the miR-130b-MV injection significantly upregulated NF-kB (p50) in both the cytoplasm and nucleus, showing enhanced proinflammation response. The above results demonstrated that miR-130b-MV activated the hepatic inflammation by inhibiting GR-mediated immunosuppression in high-fat diet-induced obese mice, suggesting a novel mechanism underlying the obesity-induced hepatic inflammation, and the inhibition of miR-130b may serve as a new molecular therapeutic target for the prevention and treatment of hepatic inflammation.

## Simple Summary

Liver diseases are clinically linked to obesity and type 2 diabetes, which have becoming increasingly prevalent worldwide. miR-130b has been previously demonstrated to regulate lipid metabolism, and plasma miR-130b is a promising biomarker for the early diagnosis and treatment of obesity. The present study aims to further determine the effect of microvesicle-shuttled miRNA-130b (miR-130b-MV) on the hepatic inflammation and its potential mechanism in high-fat diet-induced obese mice. The results showed that miR-130b-MV activated the hepatic inflammation by inhibiting GR-mediated immunosuppression in high-fat diet-induced obese mice, suggesting a novel mechanism underlying the obesity-induced hepatic inflammation, and the inhibition of miR-130b may serve as a new molecular therapeutic target for the prevention and treatment of hepatic inflammation.

## 1. Introduction

miRNAs, also referred to as microRNAs, are small non-coding RNAs that perform crucial functions in the regulation of gene expression at the post-transcriptional level [1,2]. These small molecules are approximately 20–25 nucleotides, which exhibit a high level of evolutionary conservation across different species [3,4]. Previous investigations have demonstrated that miRNAs are involved in a broad spectrum of biological processes, encompassing cellular division and proliferation, as well as the growth and maturation of organisms [5,6,7,8,9]. The exploration of miRNA expression patterns and regulatory mechanisms can furnish a more comprehensive and thorough foundation for the study and treatment of numerous diseases [10,11,12,13,14]. A large number of published results showed that miRNAs were involved in numerous diseases. In cancer, miR-155-5P can be used as a proto-oncogene, and miR-7-2 and miR-1343-3p could serve as tumor suppressor genes [15,16,17]. Most of previous studies related to miR-130b have focused on the development and progression of cancers [18,19,20] as well as lipid metabolism regulation [21,22]. In human primary preadipocytes [23], 3T3-L1 (mouse embryonic fibroblast cell line) [24] and primary cultured porcine preadipocytes [25] have demonstrated that miR-130b was able to inhibit adipogenesis. Our in vivo results have also reported that miR-130b-MV was able to decrease epididymal fat deposition through the translational repression of PPAR-γ in high-fat diet-induced obese mice [26]. However, some other reports have showed that plasma microRNA-130b (miR-130b) is a potential biomarker for the prevention and early diagnosis of obesity [24,27,28,29], suggesting that miRNA-130b is positively correlated with obesity. However, whether miRNA-130b results in hepatic inflammation needs to be further studied.

miRNAs primarily inhibit the expression of target genes by engaging in incomplete base pairing with the 3′-untranslated region (3′-UTR) of mRNAs. Using bioinformatics prediction software like TargetScan (release 7.1; http://www.targetscan.org/, accessed on September 2021), PicTar (http://pictar.mdcberlin.de/) [30], and miRanda (http://www.microrna.org/), it can be predicted that miR-130b is able to target differentially expressed mRNAs, such as PPAR-γ, PGC1α, PTEN, LDLR, PPARα, SCD-1, and GR. Among which, many predicted targets, including PPAR-γ [22], PGC1α [31], PTEN [32], low-density lipoprotein (LDL) receptor (LDLR), and the ATP-binding cassette A1 (ABCA1) cholesterol transporter [33], have been experimentally validated. It is well established that glucocorticoids (GCs) are frequently utilized in the treatment of inflammatory conditions, and the glucocorticoid receptor (GR) is their primary target for alleviating inflammation [34]. This highlights the significant role of GR in modulating immune responses and inflammation [35]. Previous research has also confirmed that GR is a potential target of miR-130b in multiple myeloma cell lines [36,37], demonstrating an important role of miR-130b in regulating the GC response. In a previous study from our research group, we demonstrated that maternal betaine supplementation during gestation significantly altered the hippocampal expression of GR as well as its regulatory miRNAs, miR-130b, and miR-181d in neonatal piglets [38]. However, according to the bioinformatic prediction results (http://www.targetscan.org), the predicted target sites (Seed sequences) for miR-130b in the 3′-UTR of GR mRNA are not highly conserved. Thus, the target relationship between GR and miR-130b remains to be verified, and whether miR-130b induces hepatic inflammation in high-fat diet-induced obese mice by inhibiting GR needs to be further studied.

Microvesicles (MVs) play a crucial role in cell communication as biomarkers and mediators and are key regulatory factors in various diseases [39,40]. Upon release from their parent cells, MVs have the ability to engage with target cells through binding to specific receptors on their membrane surfaces, or they can be engulfed by target cells. Subsequently, they discharge their contents, facilitating the transfer of genetic materials and proteins between cells for intercellular communication. MVs are packed with nucleotides (mRNA, miRNAs, et al.) and proteins, making them a significant source of various biochemical signaling molecules. This characteristic enables them to fulfill important physiological and pathophysiological functions [41,42,43,44]. Consequently, researchers have turned their attention to MVs as a novel therapeutic avenue. Numerous previous studies have shown that conjugated linoleic acid (CLA) was able to result in significant fat loss; however, these anti-obesity actions of CLA are routinely accompanied by increased markers of inflammation [45,46]. In our earlier research, we noted that miR-130b-MV effectively diminished epididymal fat accumulation by suppressing PPAR-γ in a high-fat diet-induced obese mice [26]. Additionally, another study has shown that miR-130b enhances inflammation in adipose tissue in diabetic mice by promoting M2 macrophage polarization through the inhibition of PPAR-γ [29]. These above results have suggested that miR-130b acts as a novel regulator of macrophage polarization, influencing both inflammation in adipose tissue and insulin sensitivity by suppressing PPAR-γ. Additionally, elevated levels of miR-130b were found in the bloodstream of obese individuals when compared to those with normal body weight [27]. Moreover, after TNF-α stimulation, the primary transcripts of miR-130 were significantly increased, indicating that the upregulation of miR-130 during inflammatory conditions is regulated by a transcriptional mechanism [47]. However, despite fat loss function, whether miR-130b-MV injection could inhibit GR expression and lead to hepatic inflammation is largely unknown.

The complex nuclear factor kappa-B (NF-κB) plays a crucial role in regulating the body’s innate immune system, the mammalian target of rapamycin complex, cell growth regulation, and integrated stress response maintenance. Various research efforts have demonstrated that the NF-κB signaling pathway is capable of triggering inflammation by interacting with the NOD-like receptor protein 3 (NLRP3) inflammasome and Sirtuin 1 (SIRT1), along with AMP-activated protein kinase (AMPK) [48]. The activation of NF-κB results in the expression of inflammatory mediators, such as interleukin (IL)-1β and tumor necrosis factor (TNF)-α. Previous research has also indicated that the anti-inflammatory effects of ligand-bound GR involve the binding of GR to NF-κB, which in turn inhibits NF-κB’s ability to activate transcription [49,50,51]. A specific set of genes triggered by GR, including those that work in conjunction with inflammatory transcription factors such as NF-κB, have secondary suppressive impacts on inflammatory gene expression [52]. The activation of NF-κB leads to the increased expression and enzyme activity of 11β-HSD1, which leads to increased levels of locally active GCs, and GR is an important feedforward regulator of GC activation, which increases intracellular concentrations of active GCs [52]. Locally enhanced GC activation may be necessary to subside inflammation. However, it is currently unclear whether miR-130b-MV injection could induce hepatic inflammation by suppressing GR and causing NF-κB transcriptional activation in high-fat diet-induced obese mice.

Therefore, the present study aimed to investigate the proinflammatory effect of miR-130b-MV on a high-fat diet-induced obese mice model and further clarified the potential mechanism. We demonstrated firstly that miR-130b-MV induced the hepatic inflammation by significantly increasing the expression of pro-inflammatory genes, which was closely associated with a significant downregulation of GR expression and thus NF-κB transcriptional activation. All in all, our current results provide preliminary evidence that miR-130b-MV significantly increased hepatic inflammation under obesity state, which was partly due to GR-mediated immunosuppression and NF-κB activation, suggesting a novel mechanism underlying the obesity-induced hepatic inflammation and injury as well as a novel strategy in preventive and therapeutic approaches for metabolic disorders.

## 2. Materials and Methods

### 2.1. Cells and Antibodies

The HeLa-229 human cervical cancer cell line was purchased from the Cell Resource Center of Shanghai Institute for Biological Sciences, Chinese Academy of Sciences (Shanghai, China). The antibodies used in this study are shown in Table 1 below. Synthetic RNA sequences and scrambled negative control oligonucleotides were acquired from Life Technologies Inc. (Shanghai, China).

### 2.2. miR-130b Plasmid Construction, Transfection, and MV Isolation

As we previously reported, the precursors of miR-130b (89 bp) and the negative control miRNA (scrambled control, miR-SC) were synthesized by Life Technologies Inc. based on the sequence information from miRNA precursors (www.mirbase.org) and the requirements for pSilencer 3.1-H1 siRNA expression vector (Ambion, Austin, TX, USA). The miR-130b and miR-SC plasmid construction methods could refer to our published paper [25]. HeLa-229 cells were grown in 150 mm cell culture dishes until reaching 90–95% confluence. The miR-130b and miR-SC plasmids (50 μg) were separately transfected into the cells using Lipofectamine 2000 (Life Technologies Inc.). After transfection, the cells were incubated at 37 °C with 5% CO_2_ for 4 h, and the transfection medium was replaced with DMEM/F-12 supplemented with 10% MV-free FBS, which was obtained through ultracentrifugation and filtration. After transfection for 24 h, the cells were harvested, and the culture medium was collected for MV isolation by differential centrifugation described in our previous publication [25].

### 2.3. Animals and Diets

All experiments adhered to the ethical standards for animal welfare established by the institution and were authorized by the Institute Animal Care and Use Committee at Jinling Institute of Technology located in Nanjing, China. Male SPF C57BL/6 mice, aged three weeks and weighing 9–10 g, were bought from the Comparative Medicine Center of Yangzhou University (Yangzhou, China, quality certificate: SCXK (Su) 2022-0009) and housed at the Laboratory Animal Center of Jiangsu Province Integrative Medicine Hospital in Nanjing, China.

After 7 days’ adaptation, thirty-six mice were randomly assigned to two separate groups: the control group (which received an MD10% fat diet, *n* = 12) and the high-fat diet (HFD) group (which was given an MD45% fat diet, *n* = 24). In order to avoid the oxidation of fat in feed, the feeding schedules of the two group diets were alternated every 2 days. Following an 8-week duration, the body weights of the mice were measured, and an oral glucose tolerance test was performed to confirm the successful establishment of the obese mouse model. Consequently, the successfully established obese mice were categorized into two new groups: the HF-SC-MV group (which received an injection of miR-SC-MVs) and the HF-130b-MV group (which received an injection of miR-130b-MVs). Both groups were injected via tail vein every other day for 10 days. During the 10 days of treatment, the mice in both groups were still fed an HFD.

### 2.4. Preparation of Blood and Liver Tissue

Following a 10-day treatment period, the mice underwent a fasting period lasting 10 h, during which their body weights were measured. Anesthesia for all the mice was induced via an intraperitoneal injection of sodium pentobarbital. Blood samples were collected from the abdominal aorta with a syringe. The plasma was isolated through centrifugation at a speed of 3000 rpm for 15 min at a temperature of 4 °C and subsequently stored at −20 °C for later analysis. Afterward, the mice were euthanized by cervical dislocation. The liver tissues were weighed and rapidly frozen in liquid nitrogen and then stored at −72 °C for further study.

### 2.5. Determination of Plasma Concentrations of Proinflammatory Factors IL-6 and TNF-α

The plasma concentrations of IL-6 (catalog No. 96-407) and TNF-α (catalog No. 96-422) in mice were assessed using a Luminex 200 (China Biomarker Service, catalog No. CNBMSLX200) with Magnetic Bead MAPmate assays (Merck Millipore, Darmstadt, Germany) in strict accordance with the manufacturer’s instructions.

### 2.6. mRNA Quantification

Total RNA from the liver tissue was isolated utilizing TRIzol (Life Technologies Inc.) in accordance with the manufacturer’s instructions. The concentration of the obtained RNA was measured with a NanoDrop 2000 spectrophotometer (Thermo Fisher Scientific, Waltham, MA, USA). To verify RNA integrity, denaturing agarose electrophoresis was performed, and potential DNA contamination was evaluated by PCR employing primers with the isolated RNA serving as the template. According to the protocol provided from the manufacturer (Vazyme Biotech, Nanjing, China), 1 µg of RNA was subjected to reverse transcription. The diluted cDNA (1 µL; 1:25) was used for RT-qPCR in a QuantStuio™ 6 Flex RT-PCR System (Applied Biosystems, Foster City, CA, USA). RPS17 was chosen as the reference gene to normalize the mRNA expression of the target genes, and the results were analyzed by 2^−ΔΔCT^ method. The primers used in this study were shown in Table 2.

### 2.7. miRNA RT-PCR Quantification

Firstly, the obtained total RNA was treated with RNase-free DNase I (2270A, Takara Biomedical Technology, Dalian, China) following the steps provided by the manufacturer. Next, the total RNA (8 μg) was polyadenylated by poly (A) tailing kit (EP168AF, ThermoFisher, Shanghai, China). In brief, polyadenylated RNA was obtained by reacting 8 μg of total RNA with poly (A) polymerase in a 40 μL reaction system for 1 h at 37 °C. Then, the polyadenylated RNA was then dissolved and reverse transcribed using the poly (T) adapter.

RT-PCR was performed, in triplicate, using the SYBR green qPCR master mix reagent (Takara) with a miRNA-specific forward primer and a universal reverse primer that is complementary to part of the poly (T) adapter sequence. A random DNA oligonucleotide was added to RNase-free DNase I-treated total RNA samples before polyadenylation, as an exogenous reference, to normalize the expression of miR-130b. The sequences of miRNA-130b, the poly (T) adapter, and the exogenous reference gene used in the present study are listed in Table 1.

### 2.8. Determination of Protein Content

The liver tissue was added to RIPA and PMSF for low-temperature homogenate cleavage at 4 °C. The homogenate was centrifuged at 12,000 rpm, and the separated supernatant was determined according to the manufacturer’s instructions using the BCA method (Pierce, Rockford) for protein concentration. Then, 40 μg of protein was used for 12% SDS-PAGE gel electrophoresis. Then, the protein levels were analyzed by Western blot assays. In short, electrophoretic gels were exposed by ECL after transmembrane, sealing, and incubation with antibodies. Then, Image J 1.51J8 software was used for gray analysis, with GAPDH serving as a loading control.

### 2.9. Dual-Luciferase Test

The HeLa-229 cells were seeded in 24-well plates; after reaching 90–95% confluence, the cells were transfected with pSilence 3.1-H1 neo miR-130b and pMIR-Control/GR 3′-UTR luciferase reporter plasmid, respectively. At the same time, a negative control group was set up, and the cells were transfected with pMIR-Control/GR 3′-UTR luciferase reporter plasmid and pSilence 3.1-H1 neo miR-SC. Each group had 3 replicates at least. Twenty-four hours after transfection, the cell lysate was collected after centrifugation at 12,000 rpm for 5 min, and the activity of dual luciferase was measured using Veritas Microplate Luminometer (Turner Biosystems, Sunnyvale, CA, USA) following manufacturer-provided procedures. The relative fluorescence value was measured by multifunctional enzyme marker, and the relative fluorescence activities of dual-luciferase reporter were calculated.

### 2.10. Statistical Analysis

All data are presented as the mean ± SEM (N = 12/group), and the differences between groups were analyzed using independent samples *t* tests with SPSS 20.0. The difference was regarded as statistically significant when *p* < 0.05. All the experiments were repeated at least three times.

## 3. Results

### 3.1. Body Weight, Liver Index, and Blood Proinflammation Indexes

Table 3 showed that no significant difference was observed between HF-130b-MV and HF-SC-MV mice. Nevertheless, the injection of miR-130b-MVs led to a significant decrease in body weight (*p* < 0.01) and an obvious increase in the ratio of liver weight to body weight (*p* < 0.05) when compared to the miR-SC-MV group. Additionally, the miR-130b-MV injection was associated with a minor rise in the plasma levels of IL-6 (*p* = 0.163) and TNF-α (*p* = 0.194) but without significant changes.

### 3.2. mRNA Expression of Genes Related to Hepatic Inflammation

**Figure 1:**
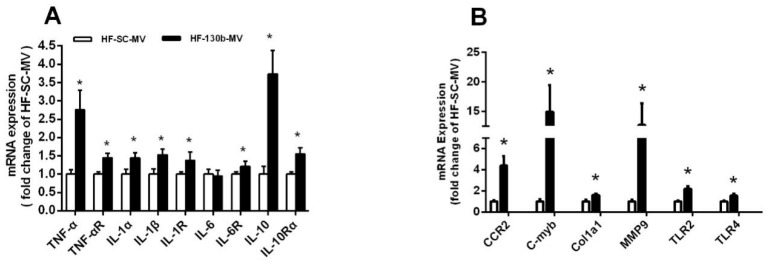
Effects of miR-130b-MV injection for 10 days on the mRNA expression of genes related to hepatic inflammation. (**A**) mRNA expression of genes related to hepatic inflammation. (**B**) mRNA expression of genes related to liver damage and inflammation. Data represent the mean ± SEM. * *p* < 0.05, compared to the HF-SC-MV group (*n* = 12).

The PCR method was used to evaluate the effect of the miR-130b-MV injection on the expression of hepatic-inflammation-related genes. The results showed that compared to miR-SC-MV-injected mice, no obvious change was observed in the expression of IL-6, while other inflammatory factors, including TNF-α, IL-1α, IL-1β, IL-10, IL-1R, IL-10R, and IL-6R (Figure 1A), as well as liver-damage-related genes CCL2 receptor (CCR2), c-Myb, col1a1, and matrix metallopeptidase 9 (MMP9) and inflammatory factors toll-like receptor 2 (TLR2) and toll-like receptor 4 (TLR4) (Figure 1B), were all significantly upregulated (*p* < 0.05) in the liver of miR-130b-MV-injected mice (Figure 1A,B).

### 3.3. Expression of miR-130b and Its Predicted Target Genes in the Liver

**Figure 2:**
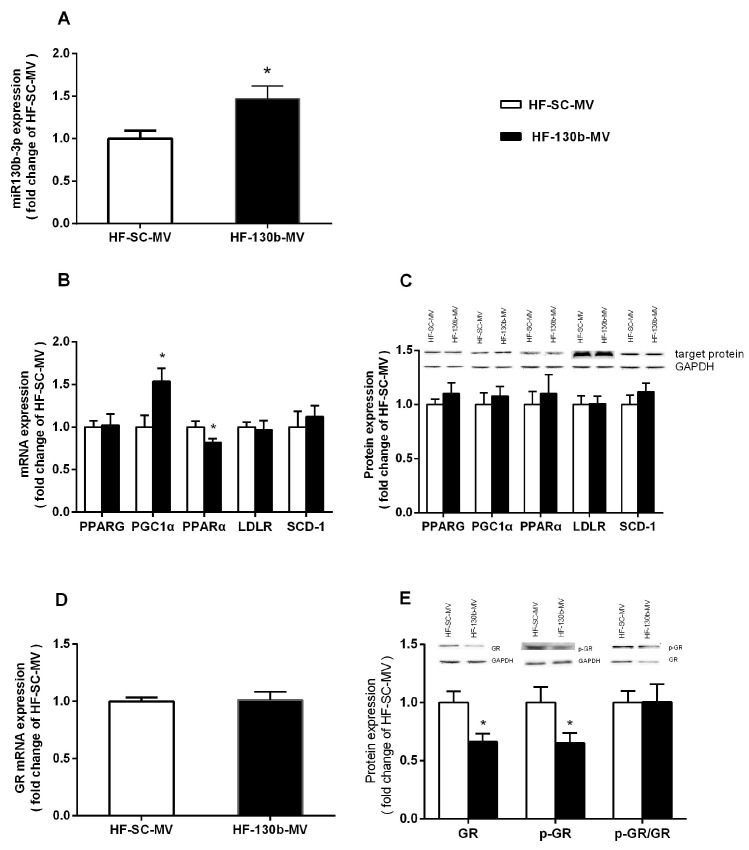
Effects of miR-130b-MV injection for 10 days on the expression of miR-130b and its target genes. (**A**) miR-130b expression. (**B**) mRNA expression of miR-130b target genes. (**C**) Protein expression of miR-130b targets. (**D**) mRNA expression of GR. (**E**) Protein expression of GR. Data represent the mean ± SEM. * *p* < 0.05, compared to the HF-SC-MV group (*n* = 12). The original images of the Western blot are published as Supplementary Materials.

As shown in Figure 2, the expression of miR-130b in the liver was significantly higher (*p* < 0.05) in the HF-130b-MV-injected group compared with the HF-SC-MV group (Figure 2A). Furthermore, PCR and Western blot methods were used to detect the mRNA expression and protein levels of the predicted target genes of miR-130b, including PPAR-γ, PGC1α, PPARα, LDLR, SCD-1 and GR, and the results showed that although the mRNA expression of PGC1α was increased and the mRNA expression of PPARα was decreased, the mRNA expression of other genes and the protein levels of all above genes were not obviously changed (Figure 2B–E) in the HF-130b-MV-injected group compared with the HF-SC-MV group. However, although the mRNA expression of GR was not changed, the protein levels of GR and p-GR were both significantly reduced (*p* < 0.05) in the liver of miR-130b-MV-injected mice compared with the miR-SC-MV-injected mice.

### 3.4. Validation of mmu-miR-130b (Mouse miR-130b) Targeting GR mRNA 3′-UTR

**Figure 3:**
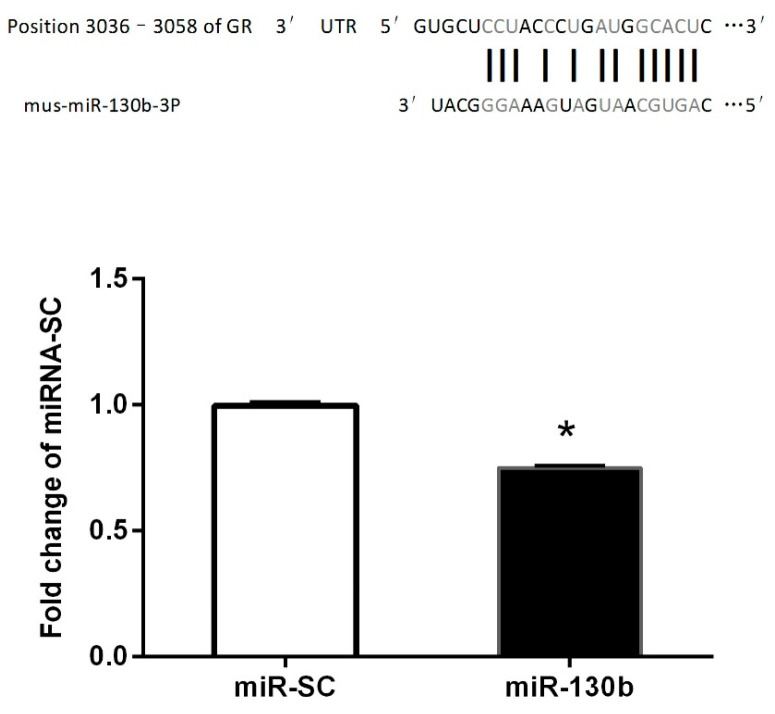
Validation of miR-130b targeting of the GR 3′-UTR. Upper panel: The single predicted binding site of miR-130b in the 3′-UTR of mouse GR. Lower panel: Activity of firefly fluorescence reporter GR 3′-UTR at 24 h after transfection of miR-130b. Data represent the mean ± SEM. * *p* < 0.05, compared to the miR-SC cells (*p* < 0.05, *n* = 3).

To ascertain whether miR-130b could recognize the GR mRNA 3′-UTR, we generated a luciferase reporter DNA construct containing the 330 bp mouse GR mRNA 3′-UTR with a putative miR-130b-binding site (Figure 3) and a mmu-miR-130b overexpression plasmid. When the pMIR-Control or pMIR-GR 3′-UTR fluorescent luciferase reporter plasmid and miR-130b overexpression vector were cotransfected into HeLa-229 cells for 24 h, the results showed that the luciferase activity was significantly suppressed in miR-130b overexpressed cells compared with miR-SC cells (Figure 3), suggesting that GR could recognize the GR mRNA 3′-UTR, and the reduced protein levels of GR and p-GR were partly due to miR-130b upregulation. These above results showed that GR is a target of miR-130b.

### 3.5. miR-130b-MVs Significantly Inhibited GR Expression and Thus Activated NF-κB Signals in the Liver

**Figure 4:**
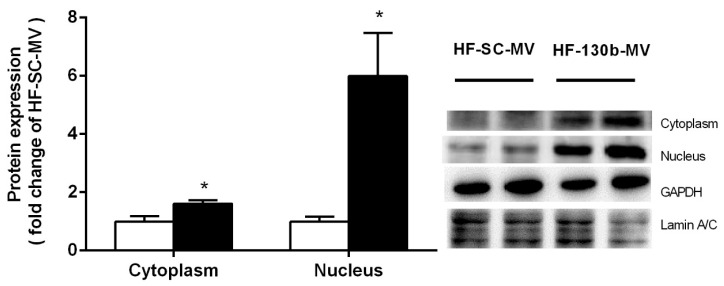
Effects of miR-130b-MV injection for 10 days on NF-kB distribution in the cytoplasm and nucleus. Data represent the mean ± SEM. * *p* < 0.05, compared to the HF-SC-MV group (*n* = 12). The original images of the Western blot are published as Supplementary Materials.

The expression of NF-κB/p50, one of the master regulators contributing to the inflammatory response, was detected by Western blot in the liver. The results showed that NF-κB expression in both the cytoplasm and nucleus was significantly increased (*p* < 0.01) in miR-130b-MV-injected mice compared to miR-SC-MV-injected mice, suggesting that miR-130b-MV significantly inhibited GR expression and thus activated NF-κB signals, which thus activated the hepatic pro-inflammation of miR-130b-MVs (Figure 4).

## 4. Discussion

In this study, feeding C57BL/6 mice with HFD for 8 weeks significantly increased the body weight by over 20%, indicating that the obese mice model was successfully established. Our previous research has shown that the miR-130b-MV injection led to a significant decrease in body weight and a reduction in epididymal fat accumulation in mice with HFD-induced obesity [26]. However, the safety of using miR-130b-MVs for treating obesity has not been thoroughly examined. Several studies have indicated that CLA was able to reduce fat accumulation similarly to miR-130b. Nevertheless, the anti-obesity effects of CLA are often associated with elevated levels of anti-inflammation markers [45,53]. Therefore, this study aimed to assess the safety of utilizing miR-130b-MVs for obesity treatment, revealing an upward trend in plasma levels of IL-6 and TNF-α after miR-130b-MV injection. Additionally, the injection of miR-130b-MVs significantly upregulated the expression of proinflammatory genes (TNF-α, IL-1α, and IL-1β and their receptors TLR2 and TLR4) and genes associated with liver damage (CCR2, c-Myb, col1a1, and MMP9) in HFD-induced obese mice, indicating that the miR-130b-MV injection led to hepatic inflammation and liver damage. This study offered the initial evidence that miR-130b-MVs could effectively upregulate the expression of proinflammation-related genes in the livers of HFD-induced obese mice.

It is well established that miRNAs play a crucial role in regulating various biological processes primarily through interaction with mRNAs. Researchers have also demonstrated that, as post-transcriptional regulators, miRNAs were able to regulate the stability and translation process of adipogenic factors and thus regulate lipid metabolism [23]. The current study focused on evaluating the expression levels of miR-130b and its putative target genes. The data demonstrated a substantial upregulation of miR-130b expression in the liver of miR-130b-MV-injected mice compared to miR-SC-MV-injected mice. This finding suggests an efficient delivery of miR-130b-MVs to the liver tissues, highlighting the superior transport capabilities of MVs as carriers of miRNAs in comparison to alternative strategies. These findings are consistent with previous research indicating successful delivery of miR-150 by MV injection, resulting in the regulation of target gene expression and functionality in recipient cells [54].

miR-130b was predicted to target differentially expressed mRNAs; therefore, we further examined the expression of genes targeting miR-130b. The results indicated that the protein level of PPAR-γ, PGC1α, PPARα, and LDLR did not change, while both GR and p-GR exhibited a significant reduction (*p* < 0.05) in the liver of mice treated with miR-130b-MVs compared to the miR-SC-MVs group. GR has been previously verified to be a target of miR-130b in multiple myeloma cell lines [36]. In this study, luciferase reporter assays were used to validate the target relationship between miR-130b and GR. The results showed that the overexpression of miR-130b in Hela-229 cells significantly inhibit the endogenous GR protein level and reduce the luciferase reporter activity of GR 3′-UTR fluorescent luciferase reporter.

GRs are able to be activated by phosphorylation, which is thus subsequently released from chaperone proteins, such as heat shock protein-90, and shift into the nucleus and perform their molecular functions. Numerous studies have indicated that GR was able to coordinate with NF-kB to regulate the expression of proinflammatory cytokines [55]. Other studies have also indicated that activated GR could inhibit pro-inflammatory factor expression [56,57,58]. The NF-κB family transcription factors are considered to be important components involved in inflammatory response, which consists of NF-κB1/p50, NF-κB2/p52, RelA/p65, RelB, and cRel [59]. The most abundant of the Rel/NF-κB dimers is the classical NF-κB p50/p65 heterodimer [60]. NF-κB, bound by the inhibitor (IκB), normally exists in an activated state as homodimers or heterodimers in the cytoplasm [61]. After translocation to the nucleus, NF-κB binds to DNA at κB sites and promotes the expression of pro-inflammatory factors [62]. Our results indicated a significant reduction in the protein content of p-GR, leading to an upregulation of the nuclear content of NF-κB (p50) in liver tissue, promoted the pro-inflammatory response in miR-130b-MV-injected mice compared to miR-SC-MV-injected mice. This suggests that miR-130b-MVs inhibit GR expression, which thus activates NF-κB signals and enhances pro-inflammatory function in the liver.

## 5. Conclusions

In summary, this study demonstrated that miR-130b-MVs could be shuttled into liver tissue, resulting in hepatic inflammation in HFD-induced obese mice, partially by inhibiting GR expression and thus activating NF-κB signals. The clarification of the potential molecular mechanism may provide a new therapeutic target for the prevention and treatment of obesity-induced hepatic inflammation.

## Figures and Tables

**Table 1:** Antibodies employed in the current investigation.

Antibody	Corporation	Catalog	Dilution
anti-GR	Santa Cruz, Dallas, TX, USA	SC1004	1:500
anti-NF-kB (p50)	Santa Cruz, Dallas, TX, USA	SC7178X	1:5000
anti-phosphorylated-GR	Cell Signaling Technology, Danvers, MA, USA	4161S	1:500
anti-LDLR	Protein Group, Qingdao, China	10785	1:500
anti-PPARG	Bioworld Technology, Shanghai, China	BS6442	1:500
anti-PGC1	Santa Cruz, Dallas, TX, USA	SC13067	1:200
anti-PPAR-α	Bioworld Technology, Dublin, OH, USA	BS1689	1:500
anti-SCD-1	Proteintech, Chicago, IL, USA	28678-1-AP	1:2000
anti-Lamin A/C	Bioworld Technology, Dublin, OH, USA	BS1446	1:500
anti-GAPDH	Minneapolis, Bloomington, MN, USA	AP0066	1:10,000

**Table 2:** Primers used in the present study.

Target Genes	Primer Sequences (5′-3′)		GenBank Accession
Plasmid construction			
miR-130b	F:GATCCGCCTGCCTGACACTCTTTCCCTGTTGCACTACTGTGGGCCACTGGGAAGCAGTGCAATGATGAAAGGGCATCAGTCAGGCTTTTTTGGAAA	R:AGCTTTTCCAAAAAAGCCTGACTGATGCCCTTTCATCATTGCACTGCTTCCCAGTGGCCCACAGTAGTGCAACAGGGAAAGAGTGTCAGGCAGGCG	MI0013136
miR-SC	F:GATCCGACTTACAGCCAGTTCCTAGTATAGTGAAGCAGCAGATGGTATACTAGGAACTGGCTGTAAGCTTTTTTTGGAAA	R:AGCTTTTCCAAAAAAAGCTTACAGCCAGTTCCTAGTATACCATCTGCTGCTTCACTATACTAGGAACTGGCTGTAAGTCG	ENSMUST00000115567.2
miR-130b expression			
miR-130b	CAGUGCAAUGAUGAAAGGGCAU	TAGAGTGAGTGTAGCGAGCA	MIMAT0013922
Exogenous reference	GTGACCCACGATGTGTATTCGC	GTGACCCACGATGTGTATTCGC	
poly(T) adapter	TAGAGTGAGTGTAGCGAGCACAGAATTAATACGACTCACTATAGGTTTTTTTTTTTTTTTTVN		
mRNA expression			
TNF-α	F: ACCACCATCAAGGACTCA	R: AGGTCTGAAGGTAGGAAGG	NM_001278601.1
IL-1α	F: AAGAAGAGACGGCTGAGT	R: GTGGTGCTGAGATAGTGTT	NM_010554.4
IL-1β	F: CTTCAGGCAGGCAGTATC	R: CAGCAGGTTATCATCATCATC	NM_008361.4
IL-6	F: CCCCAATTTCCAATGCTCTCC	R: CGCACTAGGTTTGCCGAGTA	NM_031168.2
IL-10	F:CAGTACAGCCGGGAAGACAA	R:CCTGGGGCATCACTTCTACC	NM_010548.2
IL-1R	F:GAAGGTCTTAGCTGGTGCG	R:TTCATATTCTCCTGGGCGTGC	NM_001123382.1
IL6-Rα	F:TGAATGATGACCCCAGGCAC	R:ACACCCATCCGCTCTCTACT	NM_001310676.1
IL-10Rα	F:GCCAAGCCCTTCCTATGTGT	R:CCAGGGTGAACGTTGTGAGA	NM_001324486.1
c-myb	F:GAGAGGGCCATGGGACTAGA	R:GACGTCAGCAAAGAGGGAGG	NM_001198914.1
MMP9	F: GCCGACTTTTGTGGTCTTCC	R: CTTCTCTCCCATCATCTGGGC	NM_013599.4
Col1a1	F: CGACCTCAAGATGTGCCACT	R: CCATCGGTCATGCTCTCTCC	NM_007742.4
CCR2	F: TTCTGGGCTCACTATGCTGC	R: GGCCACAGGTGTAATGGTGA	NM_009915.2
TLR2	F: ATGTTGAAGTCCAGCAGAAT	R: CCGAACCAGGAGGAAGAT	NM_011905.3
TLR4	F: TTCACCTCTGCCTTCACT	R: GGACTTCTCAACCTTCTCAA	NM_021297.3
SCD1	F: TGCCTCTTAGCCACTGAA	R: CTGTTGAGATGTGAGACTGT	NM_009127.4
PPAR-γ	F: CCACCAACTTCGGAATCA	R: GCTCTTGTGAATGGAATGTC	NM_001127330.2
PPARα	F:TTCCGTGTCAGTCTGCATCT	R:CCTGAAAGGTCCTAGCACCA	NM_011144.6
PGC1α	F:TTGACTGGCGTCATTCGGG	R:TCGCAGGCTCATTGTTGTACT	NM_008904.2
LDLR	F:CCAATCGACTCACGGGTTCA	R:CTCACACCAGTTCACCCCTC	NM_010700.3
GR	F:TGAAGCTTCGGGATGCCATT	R:ATTGTGCTGTCCTTCCACTG	NM_008173.3
RPS17	F: GGAGATCGCCATTATCCCCA	R: ATCTCCTTGGTGTCGGGATC	NM_009092.3

**Table 3:** Body weight, liver index, and blood proinflammation indexes of mice.

	HF-SC-MV	HF-130b-MV	*p* Value
liver weight (g)	1.17 ± 0.06	1.18 ± 0.05	0.85
body weight (g)	29.75 ± 0.53	26.30 ± 0.90	0.00
liver/body weight (g/100 g)	3.92 ± 0.15	4.52 ± 0.17	0.01
TNF-α (pg/mL)	6.66 ± 0.48	7.81 ± 0.69	0.19
IL-6 (pg/mL)	69.76 ± 14.10	216.99 ± 96.75	0.16

## Data Availability

The datasets used during the current study are available from the corresponding author on reasonable request.

## Supplementary Materials

The following supporting information can be downloaded at https://www.mdpi.com/article/10.3390/vetsci11110565/s1., Figure S1: The original image of the Western blot.
